# Addison’s Disease, without Organic Change in the Suprarenal Capsules

**Published:** 1865-07

**Authors:** J. F. Miner


### ART. III.—*Addison's Disease, without organic change in the Supra-renal Capsules. Report of Case.* By J. F. Miner, M. D.

Mrs. L., aged 35, was delivered of a healthy child in February last. She recovered from her confinement imperfectly, but had nursed her child and continued her usual household duties in some degree until about June 1st, five months from the time of its birth.

She had regarded herself as feeble but not sick; her appetite was pretty good; sleep comfortable, and her condition such as not to have alarmed her. At this time examination revealed the following: great emaciation, skin upon the whole surface of the body very dark, we may say in many places, black—the lighter portions being in the flexures of the joints and palms of the hands. This discoloration had come on gradually, and had now become complete, so that a lady of naturally clear and fair, though dark complexion, had become as black as a mulatto; nausea almost constant, vomiting common and very troublesome; pulse 120, and very feeble; mind clear, and spirits for the most part hopeful; respiration natural, or at least corresponded with the circulation; cough not more than had been present for years, not sufficient to attract much attention; tongue clean, surface cool; countenance remarkable, being anxious, shrunken, and indicative of serious disease; the eyes were prominent, the clear white conjunctivæ contrasting strongly with the dark color of the skin. On examining the mouth minutely, dark patches were found upon the mucous membrane, some of them half an inch in diameter; many were much smaller.

Dr. Eastman visited the patient with me while living, and in a few weeks after made *post mortem* examination in presence of a number of our professional friends, who were invited to be present on account of the rarity, interest and obscurity of such disease. The supra-renal capsules were of course the chief objects of interest and investigation; suffice it to say, however, that if they were not perfectly healthy, they were certainly not very manifestly diseased; and the *post mortem* failed to reveal any adequate cause of death.

Reports of cases of what has been called Addison's disease are common, where the supra-renal capsules are found diseased—enlarged, absent, softened or converted into tubercular masses, their fibrous envelopes thickened, or thinned—in a word, some one or a variety of diseases are found, or supposed to be found, in these glands. The theory of this disease, as proposed by Dr. Addison, would be satisfactory, or, at least, more plausible and convincing, if there was uniformly found some one change, or indeed, if in all cases any morbid change whatever could be clearly discerned. These glands are variable in size and consistence, their fibrous envelope is thicker or thinner, and the cortical or medullary structure more or less distinct, even in cases unattended by evidences of disease. It is believed that these glands may be almost entirely absent, and yet no discoloration of the skin, no anæmia, no disease; that they are changed, softened, suppurated, tubercular, or otherwise diseased in cases which are attended by discoloration of skin and great prostration, there can be no doubt. What influence these changes have in inducing such condition is not yet determined. The bronzed skin and general symptoms are observed while there is yet no discoverable change in these glands. The deposit or generation of coloring matter in the skin and mucous membrane, with the other symptoms of exhaustion and debility which mark this disease, are as yet inexplicable, though many observers have gone so far as to regard this whole matter as settled—that Addison's disease is caused by some disease of the supra-renal capsules, perhaps I should say, by *any* disease in these glands. Discoloration of the skin is quite common; pigmentary matter is deposited in patches of greater or less extent, and remains permanently, or after a time disappears. It has been attempted to connect this condition with the acute and rapidly fatal one, where the skin is bronzed and the system depressed by an influence which is certainly and often rapidly fatal. It is not known what produces the condition in either case, or what will remove it, nor is it probable that any relationship exists between them. Discoloration of the skin in pregnancy is very common, and often very annoying; still it sometimes almost entirely disappears after confinement, and is never attended by any other evidence of disease; the same thing is observed wholly disconnected with pregnancy. Bronzed skin then is not Addison's disease, nor is Addison's disease invariably attended with the cutaneous discoloration, though this must be regarded as its most important diagnostic symptom. There are great difficulties in studying carefully a disease which occurs so rarely, and this is no doubt one reason why so much remains unsettled concerning a malady, which, when fully developed has thus far proved invariably fatal. This fact alone strongly indicates that it is dependent upon organic change, and therefore included in that fatal list of diseases over which medicine has no curative influence.

Nausea and vomiting, with great prostration and debility are as essential to the disease as bronzed skin, though without the discoloration of the skin we should hardly be able to diagnose the disease even though all other symptoms were present, since the remaining indications of the malady may accompany many other affections. Neither would the bronzed skin alone show the nature of the disease, though it is the characteristic symptom of the affection which Dr. Addison has regarded as dependant upon loss of the functions of the supra-renal capsules.

There is something in the appearance of this bronzed skin which is peculiar, yet not easily described by language; when once observed it is not likely to be ever after confounded with discolorations from other causes. An inattentive and careless observer may fail at first, even in well marked cases to apprehend its nature. The case above detailed was looked upon by a former attendant and treated as jaundice; still nothing could be more distinctly not jaundice, or more certainly Addison's disease. Diagnosis cannot be difficult; it can be made quite early and positive. Treatment at whatever stage commenced is merely palative, if indeed ithas any influence whatever over its progress.

